# Household illness, poverty and physical and emotional child abuse victimisation: findings from South Africa’s first prospective cohort study

**DOI:** 10.1186/s12889-015-1792-4

**Published:** 2015-05-01

**Authors:** Franziska Meinck, Lucie D Cluver, Mark E Boyes

**Affiliations:** Centre for Evidence-Based Intervention, Department of Social Policy & Intervention, University of Oxford, Barnett House, 32 Wellington Square, Oxford, OX1 2ER UK; Department of Psychiatry and Mental Health, University of Cape Town, Cape Town, South Africa; Health Economics and HIV/AIDS Research Division, University of KwaZulu-Natal, Durban, South Africa; Health Psychology and Behavioural Medicine Research Group, School of Psychology and Speech Pathology, Curtin University, Perth, Australia

**Keywords:** Child abuse, Adolescent abuse, HIV/AIDS, Predictors, Risk factor, Chronic illness

## Abstract

**Background:**

Physical and emotional abuse of children is a large scale problem in South Africa, with severe negative outcomes for survivors. Although chronic household illness has shown to be a predictor for physical and emotional abuse, no research has thus far investigated the different pathways from household chronic illness to child abuse victimisation in South Africa.

**Methods:**

Confidential self-report questionnaires using internationally utilised measures were completed by children aged 10-17 (n = 3515, 56.7% female) using door-to-door sampling in randomly selected areas in rural and urban locations of South Africa. Follow-up surveys were conducted a year later (96.7% retention rate). Using multiple mediation analyses, this study investigated direct and indirect effects of chronic household illness (AIDS or other illness) on frequent (monthly) physical and emotional abuse victimisation with poverty and extent of the ill person’s disability as hypothesised mediators.

**Results:**

For children in *AIDS-ill families*, a positive direct effect on physical abuse was obtained. In addition, positive indirect effects through poverty and disability were established. For boys, a positive direct and indirect effect of AIDS-illness on emotional abuse through poverty were detected. For girls, a positive indirect effect through poverty was observed. For children in *households with other chronic illness*, a negative indirect effect on physical abuse was obtained. In addition, a negative indirect effect through poverty and positive indirect effect through disability was established. For boys, positive and negative indirect effects through poverty and disability were found respectively. For girls, a negative indirect effect through poverty was observed.

**Conclusions:**

These results indicate that children in families affected by AIDS-illness are at higher risk of child abuse victimisation, and this risk is mediated by higher levels of poverty and disability. Children affected by other chronic illness are at lower risk for abuse victimisation unless they are subject to higher levels of household disability. Interventions aiming to reduce poverty and increase family support may help prevent child abuse in families experiencing illness in South Africa.

**Electronic supplementary material:**

The online version of this article (doi:10.1186/s12889-015-1792-4) contains supplementary material, which is available to authorized users.

## Background

Approximately 40 million children under 14 years of age are victims of abuse and neglect worldwide [1], with children in the sub-Saharan African region suffering from particularly high rates of abuse [2,3]. Explanations for these elevated prevalence rates in Africa often lack empirical basis. Poorly developed child protective systems, modernisation and negation of traditional values, large numbers of orphaned children, and disruption of community structures and social norms are some hypothesised causes [4].

### Illness and abuse

Like other countries in the region, South Africa is also experiencing a considerable burden of disease, with large numbers of people suffering from communicable (e.g. HIV or TB) and non-communicable illnesses (e.g. high blood pressure or diabetes) [5]. Research has shown that violence and poor health are correlated, especially in low and middle-income countries in Africa [6] and a recent systematic review of correlates of child abuse victimisation in Africa found an association between household illness and child maltreatment^a^ [7]. It is, however, unclear whether the cross-sectional relationship between household illness and child abuse is sustained over time using longitudinal data. No research has thus far examined whether households with certain types of chronic illnesses such as those related to AIDS differ in their risk for physical and emotional child abuse victimization. This may be due to specific direct and indirect pathways from household chronic illness to challenges in parenting within the home.

### Investigating pathways to abuse

In order to understand the relationships between household illness and child abuse, it can be valuable to situate household illness within a larger ecological model [8]. This framework places the child at the centre of multiple interacting spheres of influence such as peers, family, community and society. While there may be a direct effect of household illness on child abuse, an indirect effect of household illness on risk for child abuse victimisation through additional factors (i.e. stress, pain, fatigue or stigma) is probable [9].

Indirect effects of household illness on risk for child abuse victimisation are investigated using mediators. Mediating factors are variables which play an important role in governing the relationship between the hypothesised risk factor and outcome. As chronic illness can affect patients differently and manifest in different ways, some aspects of suffering from a chronic illness may be particularly prone to affect the risk for child abuse victimisation (i.e. poor mental health), while others are not. Mediation analysis can be used to examine how these particular aspects influence the relationship between chronic illness and child abuse. Whether or not there is a direct link between chronic illness and child abuse victimisation, it is possible that chronic illness exacerbates other factors which in turn increase the risk for child maltreatment.

### Linkages between poverty, disability, ill health and abuse

A recent systematic review identified household poverty and disability as common correlates of physical and emotional child abuse victimisation in Africa [7]. International research has found strong bidirectional positive linkages between poverty and ill health [10,11] and positive correlations between child maltreatment, poverty and caregiver ill health [12,13]. A trial of an intervention carried out in Wisconsin with families affected by poverty showed lower likelihood of child protection investigations in families who received more financial support compared to those who received less [14]. Previous studies in South Africa have found that children in AIDS-affected families report consistently higher levels of poverty than children in healthy or other ill families [15,16]. Studies from the United Kingdom suggest that parents with disabilities are more likely to live in low-income households and to be economically inactive [17]. In addition, parents or caregivers with disabilities may be at greater financial disadvantage because they have to pay for additional support in and outside the household and while parenting [18].

Studies from the United States found that mothers with chronic pain reported more laissez-faire parenting and poorer relationships with their children [19]. They experienced more psychosocial distress, which impacted on their parenting, in particular their ability to parent positively [19,20]. Likewise, paternal illness predicted negative family functioning [21] The larger the number of stress factors (such as ill health, physical problems and poverty) a parent experienced, the less likely they were to cope with parenting stress [22].

However, existing evidence is thus far unclear about the ways in which household illness, poverty, and disability link together with child abuse victimisation. A previous cross-sectional study from South Africa found that caregiver AIDS-illness was linked to poverty, extent of disability of the ill person and abuse [15]. Evidence suggests that particularly families affected by AIDS appear to be at higher risk for child maltreatment [23], and that those affected by other chronic illness have lower or equal risk to those in healthy families [13]. It is unclear, however, what the mechanisms of these relationships are.

### Poverty and disability as potential mediators between household illness and abuse

There are a number of routes by which poverty may be increasing risk of abuse. Previous research suggests that economic status and social support are highly correlated with caregiver depression [24] which is further exacerbated by food insecurity [25]. Poverty and poor physical health also predict increased psychological stress in kinship carers [26]. In settings with high HIV-prevalence, high levels of stress, depression and poor physical health were found amongst adults caring for children [27]. A previous study from South Africa found that household AIDS-illness was associated with reduced capacity of positive parenting [28]. Research from high-income countries also shows poorer parent-child relationships and more inconsistent parenting amongst HIV positive parents and those suffering from chronic pain [21,29,30].

There are also number of pathways in which disability may be increasing risk of abuse. Previous research suggests that physical disability in chronically ill patients is highly correlated with poor mental health [31]. A larger number of symptoms and higher extent of disability was found to be associated with higher parental distress and aggravation during parenting tasks [32]. Furthermore, illness-related demands predicted lower parenting quality, which in turn predicted child behaviour problems [33]. Household illness and high levels of disability, coupled with stigma and poverty may therefore lead to increased stress, poorer parenting and mental health, which can increase child conduct problems, all of which have been found to be risk factors for child maltreatment [12].

To date, the understanding of risk factors for child maltreatment in South Africa has been limited. First, all of the studies to date are cross-sectional in design which limits determination of directions of association [7,34]. Second, the majority of studies used retrospective recollections of childhood abuse [35,36], which may be subject to recall bias [37]. Third, samples mostly consist of high-school and university students [38,39], which may exclude some of the most vulnerable children who may not be reaching these levels of education [23]. Fourth, some studies use patient chart data from mental health units, court records and social services [40,41] that are subject to bias as only the most severe or most identifiable cases may have been reported to officials [42]. Fifth, no published study has examined direct and indirect pathways from household chronic illness to risk for child abuse victimisation.

**Figure 1:**
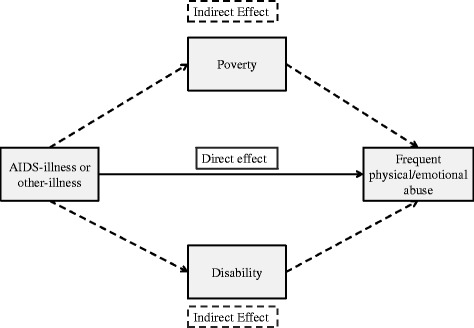
Hypothesised direct effect and partial indirect effects of household chronic illness on physical and emotional abuse.

The current study therefore had two aims. First, we aimed to determine the direct and indirect effects of baseline household *AIDS-illness* on physical and emotional child abuse victimisation at follow-up. Second, we aimed to examine direct and indirect effects of baseline *other household chronic illness* on physical and emotional child abuse victimisation at follow-up (Figure 1). Analyses were conducted separately for boys and girls for emotional abuse due to significant differences in victimisation between the genders.

## Methods

### Participants

3515 children aged 10-17 (mean age 13.5 years, 56.7% female, 50.6% urban location) were originally recruited between January 2010 and June 2011 in four health districts with >30% HIV prevalence in rural and urban areas of Mpumalanga and the Western Cape. Within each health district, census enumeration areas were randomly selected. All households with children aged 10-17 within each census enumeration area were included in the study. One child in each household was interviewed and where there were multiple children in the household, one was chosen at random. Between January 2011 and June 2012, 3401 participants (96.7% retention rate) were traced and re-interviewed. Refusal rate at baseline was 2.8% and < .5% at follow-up. Adults were not recruited for this study but had to give consent for their child’s participation.

### Procedure

With interviewers, children completed an anonymous guided 60-minute self-report questionnaire which was translated into Xhosa, Swati, Tsonga, Sepedi and Zulu and checked by back-translation. Interviews were carried out in locations selected by the child in order to guarantee confidentiality and privacy (e.g. under a secluded tree, empty classrooms). Interviewers from the area received intensive training in working with vulnerable children and in administering standardised questionnaires. Participation was voluntary and children were able to stop the interview at any time. All participants received a certificate of appreciation for taking part in the research and light refreshments irrespective of completion of the questionnaire. Certificates showcased international celebrities on the front and contained information about the complaints procedure of the study and contact details for telephone helplines such as Childline, Lovelife and the local police on the back.

Due to low literacy in the sampled population group, information and consent sheets were read aloud to children and their caregivers and clarification questions answered until participants were satisfied and consented to take part. Stringent quality checks were in place so that missing data were < .05%. All survey items were pre-piloted with vulnerable youth to investigate age appropriateness and cultural sensitivity. Ethical approval was granted by the University of Oxford, University of KwaZulu-Natal, University of Cape Town, Provincial Departments of Health and Education and the National Department of Social Development.

Confidentiality was maintained throughout the study unless participants were considered to be at risk of significant harm or requested help, and this was clearly outlined in the consent forms. Where this was the case, the project manager and interviewer discussed options of referrals with the child. Immediate referrals were made following discussion with participants to local child protection services for children experiencing ongoing severe abuse. Where children had experienced abuse in the past, referrals to counselling centres and HIV-testing services were made where appropriate and requested. 145 referrals were made at baseline, 664 referrals were made at follow-up.

#### Measures

All measures of abuse were pre-piloted and modified to fit the cultural context with the help of experienced social workers, child protection NGOs and vulnerable children in South Africa*.* The whole abuse scale showed good reliability in this sample (α = .73). *Child physical and emotional abuse victimisation* at follow-up were measured using seven items from the UNICEF Measures for National-Level Monitoring of Orphans and other Vulnerable Children [43] that are based on the Conflict Tactic Scales for Parent and Child (CTSPC) [44]. The CTSPC has been used in international studies across the world [45,46]. The UNICEF measure has not been validated in South Africa but was successfully used in another study in the Western Cape with good reliability (α = .70) [23]. Seven additional items were devised through qualitative pre-piloting with practitioners and vulnerable children (α = .74 for all 14 items on this subscale). Past-year frequency of abuse was measured (*0: never; 1: not in the last year; 2: at least once this year; 3: month; 4: weekly*). A conservative threshold for frequent abuse was set as occurrence of physical or emotional abuse on a monthly or more frequent basis within the last year (see Additional file 1 for complete list of items and Additional file 2 for the original response values) and a dichotomous variable was created for physical and emotional abuse respectively (*0: no monthly abuse; 1: yes monthly abuse*).

*Household chronic illness and extent of disability* were measured using a Verbal Autopsy Checklist [47], which included symptoms of AIDS-related and other chronic illnesses common in South Africa such as diabetes, high blood pressure, arthritis, alcoholism, emotional problems, and cancer [48]. The Verbal Autopsy has been validated in South Africa [49] and was applied to all household members who had been ill for a period of at least two weeks. Determination of household AIDS-illness required identification of three or more AIDS-defining illnesses (i.e. HIV-wasting syndrome, Kaposi sarcoma, oral candidiasis, vaginal cancer, jaundice or herpes zoster). Dichotomous variables were created for household AIDS-illness (*0: not ill with AIDS; 1: ill with AIDS*) and other chronic illness (*0: not ill with other chronic illness; 1: ill with other chronic illness*). Extent of the ill person’s disability was measured using 7-items from the WHO International Classification of Functioning, Disability and Health ‘activity limitation and participation’ sub-scale [50]. Example items include difficulty of carrying shopping or carrying out personal hygiene, and are responded to according to level of difficulty (*0: not at all difficult; 1: a little difficult; 2: very difficult; 3: not able to do it*). Items were summed to give a total disability score. The scale showed good reliability in this sample α = .93.

*Household poverty* was measured using an index of access to the eight highest socially-perceived necessities for children in South Africa [51], which showed good reliability of α = .80 in this sample. Necessities included: enough clothes to remain warm and dry, soap to wash every day, three meals per day, a visit to the doctor and medicines when needed, school uniform, money for school fees and more than one pair of shoes. Items were reverse scored (*0: has access to item; 1: does not have access to item*) and summed to give a total poverty score (i.e. total number of necessities lacking).

*Demographic covariates* of gender, age, receipt of pension, formal/informal housing and urban/rural location were measured using items modelled on the South African Census [52].

##### Analysis

Analyses were conducted in three stages, using SPSS 20. First, differences in socio-demographic characteristics and physical and emotional abuse victimisation between children lost (n = 114) and retained (n = 3401) at follow-up were investigated. Second, descriptive analyses and comparison of means (ANOVA) investigating relationships between gender, illness-status, disability and poverty were carried out. Third, multiple mediation tests using OLS for and logistic regression analyses for dichotomous outcomes with the PROCESS macro [53] were conducted to determine direct and indirect effects of chronic household illness on child abuse victimisation. Other than Baron & Kenny [54], Hayes [55] and Zhao, Lynch, & Chen [56] do not require that two variables have to be associated with each other in order to test hypotheses of indirect effects.

Multiple mediation analyses used Preacher and Hayes’ [57] bootstrapping procedure. This is a nonparametric sampling procedure recommended for simultaneous testing for indirect effects of multiple mediators [58]. It allows determination of the extent to which each mediator variable affects the relationship between the hypothesised predictor and the outcome in the presence of other potential mediators. Tests for significant mediation required bias-corrected 95% confidence intervals to not overlap zero, based on 1000 bootstrap samples. Mediation analyses investigating emotional abuse were conducted separately for boys and girls considering a higher risk for emotional abuse and higher prevalence rates of family AIDS in girls ([23], Table 1). Analyses investigating physical abuse adjusted for gender. Existing evidence suggests a direct effect of family AIDS on physical and emotional abuse victimisation and no direct effect of other chronic illness [13]. Therefore, analyses were conducted separately for children affected by AIDS and those affected by other-illness. All mediational analyses adjusted for age, rural/urban location, informal housing, province, receipt of pension, and made use of the temporal order within the data: predictors and mediators were measured at baseline, outcomes measured at follow-up.

**Table 1:** **Gender differences in the variables used for analysis**

	**Boys (n = 1475)**	**Girls (n = 1926)**
Rural area at baseline	42.4% (712)	57.6% (969)
Mpumalanga province at baseline	45.3% (746)	54.7% (902)*
Informal housing at baseline	41.6% (444)	58.4% (624)*
Mean age at baseline	13.41 (SD 2.10) SE .055	13.44 (SD 2.18) SE .050
Poverty at baseline	2.61 (SD 2.30) SE .060	2.74 (SD 2.33) SE .053
Household receipt of pension at baseline	13.2% (194)	13.0% (251)
Frequent physical abuse at follow-up	44.5% (251)	55.5% (313)
Frequent emotional abuse at follow-up	38.5% (271)	61.5% (433)**
Frequent physical abuse at baseline	17.0% (251)	19.1% (368)
Frequent emotional abuse at baseline	17.4% (257)	20.6% (397)*
AIDS-illness at baseline	39.2% (417)	60.8% (646)***
Other chronic illness at baseline	46.3% (219)	53.7% (254)
Extent of ill person’s disability at baseline	1.97 (SD 3.87) SE .101	2.14 (SD 3.80) SE .087

*Chi* ^*2*^ *and two-sample t-tests Note:* *p < .05, **p < .01, ***p < .001.

## Results

### Children lost and retained at follow-up

Children lost to follow-up did not differ from those retained with regard to gender (*χ*^2^ = 0.07; p = 0.789) or frequent physical abuse (*χ*^2^ = 1.562; *p =* 0.211). However, children lost at follow-up were more likely to have experienced frequent emotional abuse (*χ*^2^ = 6.624; *p =* 0.010), were older (*t* = 6.44; *p* = 0.011), and lived in poorer households (*t* = 21.55; *p <* 0.001) than those retained. It is therefore possible that more vulnerable children were lost to follow-up and findings should be interpreted with this in mind.

Socio-demographic statistics for the population sample are summarized in Table 2. The sample included 1095 participants from AIDS-ill households (31.2%) and 482 participants from households with other chronic illness (13.7%) at baseline. Girls had higher rates of emotional abuse (*χ*^2^ = 8.591; *p =* 0.003) and living in an AIDS-ill household (*χ*^2^ = 11.061; *p =* 0.004) (Table 1). Households affected by AIDS were experiencing higher levels of poverty and disability compared to those with other chronic illnesses and healthy households. Households affected by AIDS had significantly higher prevalence rates for physical and emotional abuse compared to healthy households. Those affected by other chronic illness had lower prevalence rates of abuse than healthy households (Table 3). Prevalence rates in this study were 16.6% for frequent physical and 20.7% for frequent emotional abuse victimisation. The relationship between the interviewed child and the ill person within the household was as follows: 40.2% were mothers, 24.2% grandparents, 11.9% fathers, 8% siblings, 3.2% the respondent themselves, 11.7% other family members, 0.4% non-relatives and 0.3% foster parents.

**Table 2:** **Characteristics of the sample at baseline and follow-up**

	**Baseline (n = 3515)**	**Follow-up (n = 3401)**
Gender (female)	56.7% (1992)	56.6% (1926)
Rural area	49.4% (1737)	49.4% (1681)
Mpumalanga Province	47.3% (1664)	49.8% (1681)
Informal housing	31.8% (1117)	20.6% (701)
Mean age	13.45 years (SD 2.15) SE .036	14.67 years (SD 2.22) SE .038
Poverty	2.71 (SD 2.32) SE .040	2.75 (SD 2.34) SE .040
Household receipt of pension	13.1% (459)	9.2% (314)
Frequent physical abuse	18.3% (645)	16.6% (564)
Frequent emotional abuse	19.5% (687)	20.7% (704)
AIDS-illness	31.2% (1095)	17.7% (602)
Other chronic illness	13.7% (482)	12.5% (424)
Extent of ill person’s disability	2.08 (SD 3.87) SE .065	1.18 (SD 3.08) SE .053

**Table 3:** **Baseline characteristics of the outcome and mediator variables split by household illness status**

	**Healthy (comparison group) (n = 1824)**	**Other chronic illness (n = 482)**	**AIDS-ill (n = 1095)**
Physical abuse at follow-up	14.9% (278)	11.3% (64)*	20.9% (222)*
Emotional abuse at follow-up	18.7% (349)	19.9% (94)	24.6% (261)*
Poverty at baseline	2.58 (SD 2.29) SE .05	2.06 (SD 2.02) SE .93*	3.14 (SD 2.39) SE .07*
Disability at baseline	.36 (SD 1.76) SE .04	2.81 (SD 3.87) SE .18*	4.74 (SD 4.73) SE .15*

*Chi* ^*2*^ *and One-Way-Anova tests. Note:* *p < .05.

### Mediation analysis

Mediational analyses were conducted for both household AIDS-illness and household other chronic illness in line with Hayes [46] to establish the extent of mediation. Six separate models tested the direct and indirect effects of household AIDS-illness and other chronic illness at baseline on child abuse at follow-up through poverty and the extent of the ill person’s disability at baseline. Models were run separately for boys and girls for emotional abuse. All analyses controlled for age, rural location, informal housing, receipt of pension and province.

### Frequent physical abuse victimisation

**Figure 2:**
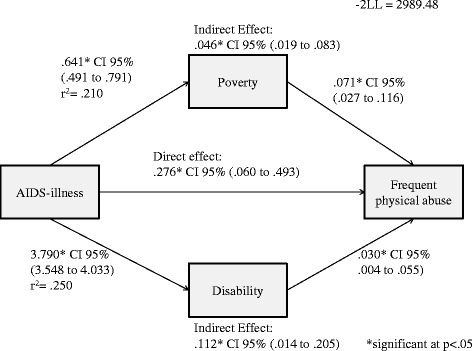
Direct and indirect effects of AIDS-illness on frequent physical abuse.

A positive direct effect of household AIDS-illness on frequent physical abuse victimisation was observed (*B =* 0.276, 95% *CI* 0.060 − 0.493). Additionally, a positive indirect effect of household AIDS-illness on frequent physical abuse through poverty (*B =* 0.046, 95% *CI* 0.019 − 0.083) and disability (*B =* 0.112, 95% *CI* 0.014 − 0.205) was obtained (Figure 2).

**Figure 3:**
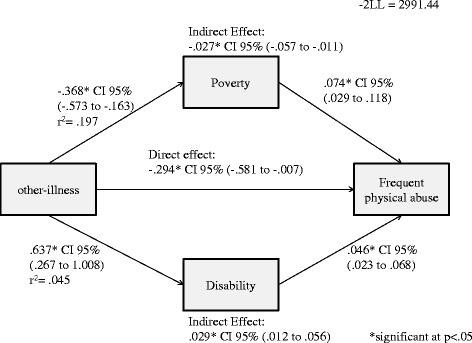
Direct and indirect effects of household other-illness on physical abuse.

A negative direct effect of other chronic illness on frequent physical abuse victimisation was observed (*B =* -0.294, 95% *CI* -0.581 − -0.007) was observed. Additionally, a negative and positive indirect effect of household other chronic illness on frequent physical abuse victimisation through poverty (*B =* -0.027, 95% *CI* -0.057 − -0.011) and disability (*B =* 0.029, 95% *CI* 0.012 − 0.056) were obtained respectively (Figure 3).

### Frequent emotional abuse victimisation

**Figure 4:**
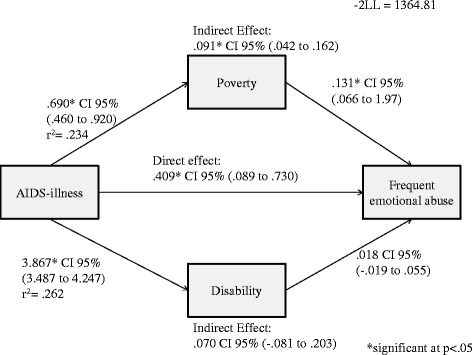
Direct and indirect effect of household AIDS-illness on frequent emotional abuse in boys.

For boys (Figure 4), a positive direct effect of household AIDS-illness on frequent emotional abuse victimisation was observed (*B =* 0.409, 95% *CI* 0.089 − 0.730). Additionally, a negative indirect effect of household AIDS-illness on frequent emotional abuse through poverty was obtained (*B =* 0.091, 95% *CI* 0.042 − 0.162). Disability did not affect the relationship between AIDS-illness and frequent emotional abuse.

**Figure 5:**
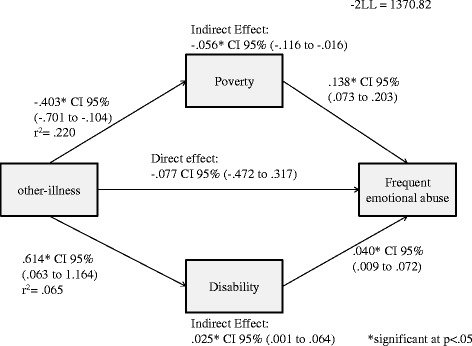
Direct and indirect effects of household other-illness on frequent emotional abuse in boys.

There was no direct effect of other chronic illness on frequent emotional abuse. However, a negative and positive indirect effect of other chronic illness on frequent emotional abuse through poverty (*B =* -0.056, 95% *CI* -0.116 − -0.016) and disability (*B =* 0.025, 95% *CI* 0.001 − 0.064) respectively was observed (Figure 5).

**Figure 6:**
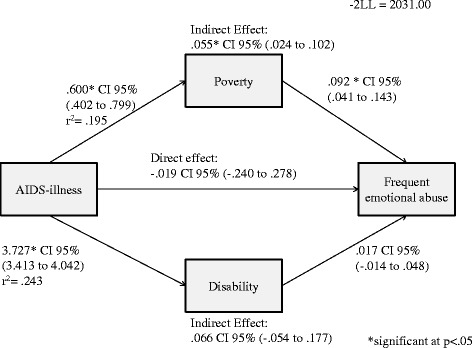
Direct and indirect effect of household AIDS-illness on frequent emotional abuse in girls.

For girls (Figure 6), a positive indirect effect between household AIDS-illness and frequent emotional abuse victimisation through poverty (*B =* 0.055, 95% *CI* 0.024 − 0.102) was observed. Disability did not affect the relationship between AIDS-illness and frequent emotional abuse.

**Figure 7:**
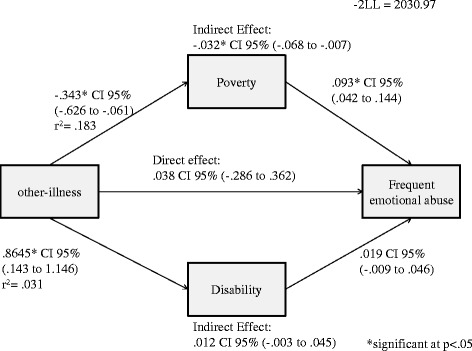
Direct and indirect effect of household other-illness on frequent emotional abuse in girls.

There was no direct effect of other chronic illness on frequent emotional abuse. However, a negative indirect effect of other chronic illness and frequent emotional abuse through poverty (*B =* -0.032, 95% *CI* -0.068 − -0.007) was observed (Figure 7).

## Discussion

This is the first large-scale longitudinal study examining the pathways from household chronic illness to child abuse in the developing world through multiple mediation analysis. AIDS-affected households, showed higher levels of physical and emotional abuse compared to healthy households while households affected by other chronic illness had lower abuse prevalence rates.

There are no research findings to date that explain the difference in abuse risk between families affected by AIDS and those affected by other chronic illness. It is possible that families affected by AIDS experience additional and different stress factors to families affected by other chronic illness. These could be fear of death and severe symptomology [59], AIDS-related stigma [60] and lower quality of life [61]. Furthermore, families affected by chronic illness in this study suffered from diseases with more straightforward treatment options and lower perceived stigma such as diabetes or high blood pressure. Existing studies investigating parenting in families with chronic illness either focused on AIDS-affected or cancer-affected families [19,21], both illnesses with high levels of stigma and perceived shorter life expectancy. Results from previous studies might therefore be more applicable to AIDS-affected families than to those affected by other chronic illness. Future research could valuably explore linkages and differences between these factors.

Direct and indirect effects of household chronic illness on physical and emotional abuse victimisation were found. In particular, direct and indirect effects were observed for household AIDS-illness showing increased risk for abuse victimization for children in AIDS-ill families through poverty and disability. Direct and indirect effects were also found for households with other chronic illness, surprisingly, showing reduced risk of abuse and poverty for children in other ill households. However, an increased risk for severe disability taht increased risk for child abuse victimisation was also observed. The findings of this study therefore extend previous research from South Africa which found direct associations between physical and emotional abuse victimisation and household AIDS-illness but not with other chronic illnesses [13,23] and the findings partially correspond with this.

As hypothesised, disability was an important mediator of the relationship between household AIDS-illness and physical abuse, but surprisingly not emotional abuse. Furthermore disability mediated the relationship between other-chronic illness and physical abuse for the whole sample and emotional abuse for boys only. The role of disability as a mediator is consistent with research linking poor caregiver health to higher risk of abuse [21].

Poverty was also an important mediator of the relationship between AIDS-illness and physical and emotional abuse. Unexpectedly, lower levels of poverty were a protective mediator of the relationship between other chronic illness and physical and emotional abuse. This study therefore corroborates current evidence that found that households affected by other chronic illness in South Africa appear to have a lower risk for poverty compared to healthy and AIDS-affected ones [62]. The lower risk for poverty in households affected by other chronic illness in this current study decreased the risk for child abuse victimisation in the mediation models. However, poverty as a factor itself remained clearly linked to an increased risk of child abuse.

Differences in poverty risk between households affected by AIDS and other chronic illness could be attributed to differences in the age groups between the ill household members. Chronic illnesses measured (i.e. high blood pressure, diabetes) may be more likely to appear in older age people who are entitled to a state pension in South Africa [63]. Of the children in households with chronic illness in this study, 26.6% reported being cared for by their grandparents compared to 13.8% in AIDS-affected households. State pensions have been shown to reduce household poverty as they are spread across all members of a household [64,65].

The role of poverty is consistent with research linking AIDS-affected households with high levels of deprivation [66] as AIDS-illness increases household poverty [67] through inability to work, medical expenses and excessive funeral costs in case of AIDS-death [68]. On the other hand, poorer households are at higher risk for HIV infection and this can set up a vicious cycle [69].

Considerable differences were found in the pathways to abuse depending on the abuse outcome, the child’s gender and the illness status of the family. Differences in maltreatment according to the child’s gender and to family illness were expected due to previous studies suggesting differences in risk between these groups [13,70]. However, no previous research has investigated pathways from illness to abuse in a similar fashion before, and speculation about these differences in results would go beyond the scope of the data. Thorough future research is needed to corroborate these findings and examine possible reasons for these differences. If these persist in future studies, there may be implications for policy makers and practitioners in focussing interventions.

### Limitations and future research

This study had a number of limitations. First, less than two-thirds of the South African population know their HIV status, which makes self-reporting of HIV status unreliable [71]. This study was therefore not able to identify households with HIV+, but asymptomatic members. However, the verbal autopsy to identify AIDS-illness has been successfully used in previous studies with good reliability [15,23]. Furthermore, identifying only households with AIDS sequelae allows for a fuller understanding of this subgroup of individuals. Second, no scales for child abuse victimisation have been validated for use in South Africa. However, all scales were successfully used in prior studies and showed good reliability in this sample [13,23].

Third, the study was carried out in randomly sampled areas with 30%+ HIV prevalence. Results are therefore not generalisable across the South African child population but give a good indication of risks for children in low-income areas with high HIV prevalence. Fourth, this study measured the risk of abuse in families affected by chronic illness, however, it should be noted that the ill person and the person abusing the child may not be one and the same. However, the results clearly indicate that household illness increases the risk for child abuse victimisation through poverty and extent of disability.

Fifth, referrals to child protective services at baseline could have potentially influenced the results and levels of abuse at follow-up. Unfortunately, social services in South Africa are overburdened and understaffed and rarely able to respond to referrals in a timely manner [72]. Only a tiny number of children referred at baseline (<3%) had been contacted by the appropriate services by follow-up and impact of baseline referrals on results is therefore unlikely. Sixth, the study measured child abuse committed by an adult within the child’s network but investigated mediation between household factors. The perpetrator could, therefore be an adult outside the child’s home i.e. a teacher. In this study, 74.6% of all physically and emotionally abusive acts were carried out within the child’s home, with parents and relatives as the perpetrators [73], suggesting that the observed effects on physical and emotional abuse are primarily associated with events occurring within the household.

Seventh, the data presented cannot determine causality and this study was therefore not able to determine whether living in a family affected by chronic illness causes an increased risk for child abuse victimisation. Longitudinal observational designs allow for controlling of baseline confounders and identification of correlate directionality because the hypothesised risk factors precede the outcome [34]. They are therefore superior to cross-sectional studies where temporality cannot be determined. This study established that baseline chronic illness has an effect on risk for child abuse victimisation at follow-up. Temporal order could not be established for the mediation analyses as these were only cross-sectional due to only two time points collected. However, cross-sectional mediation analyses can be used for theory generation and development, with the understanding that the hypothesis arising from these analyses will then have to be verified in longitudinal data [74].

Eighth, there is a strong likelihood of unmeasured confounding in this study as suggested by the low values in R^2^. Even though models adjusted for potential confounding variables reported by children, caregiver related confounders such as mental health or substance use could not be accounted for. Due to the design of the study, unmeasured confounding cannot be ruled out.

Finally, the study used child self-report with interviewer-guided questionnaires. Opinions differ whether children are reliable informants regarding disability and illness within the household. However, previous research has used the verbal autopsy and disability measures successfully [15] and has shown that children often carry out caring tasks within the home that allow them to witness physical ability and symptomology of ill household members [75]. Furthermore, a recent study investigating inter-rater reliability between adult-child dyads using the verbal autopsy tool found concordant reporting of adult HIV status to be 72% and no significant association between concordance and child age [76].

Interviewer presence during surveying may have increased the likelihood of under-reporting, in particular of socially undesirable events such as child abuse. Computer assisted interviewing may increase reporting of stigmatized events or behaviours in some cases [77]. However, it may not be suitable for all settings, such as the very rural ones in which parts of this study were conducted and where participants may be intimidated by the opportunity to use a computer to answer questions [78,79]. The advantage of the system used in this study is that it allowed for more detailed answers and a very good interviewer-participant relationship, which facilitated follow-up. Future work is needed to examine other potential factors, such as parental risk factors of mental health and substance abuse [12,80,81] and predictors of multiple abuse victimisation.

## Conclusions

This is the first study to investigate pathways from household illness to physical and emotional child abuse via poverty and disability. There are currently an estimated 85 million AIDS-affected children in sub-Saharan Africa [82] and millions more in households affected by chronic illness [83]. The present study highlights the differences in risk for child maltreatment in families affected by AIDS and those affected by other chronic illness, with those affected by AIDS at higher risk for physical and emotional abuse victimisation. Findings showed that pathways to abuse operated differently and even contradictory depending on family illness status. They suggest the importance of recognising two groups of children at heightened risk of child maltreatment: AIDS-affected and those affected by other chronic illness with high levels of disability. Services should include this in assessments of child well-being. In particular, interventions that effectively lower household poverty levels and support families with chronic illnesses may have additional positive impacts on reducing risks of child maltreatment, although further research is essential to confirm these findings.

In South Africa, social grants have been found to be effective in reducing household poverty and improving child outcomes [84,85]. Another effective way to support caregivers and reduce abuse are parenting interventions [86], and while there is limited evidence for these from South Africa and other low- and middle-income countries [87], a suite of parenting interventions for this purpose and various age groups is currently being tested [88]. In order to reduce the compound vulnerability of children in households affected by chronic illness and to address child abuse in South Africa, it is essential that we rigorously test child abuse interventions and take those that are effective to scale.

### Endnote

^a^For the purpose of this paper child abuse and child maltreatment will be used interchangeably.

## Additional files

Additional file 1:
**Original measurement items for child physical and emotional abuse victimisation.**
Additional file 2:
**Physical and emotional abuse at follow-up: Individual response frequencies.**
