# Supplementary material for: Household illness, poverty and physical and emotional child abuse victimisation: findings from South Africa’s first prospective cohort study
Source: BMC Public Health. 2015 May 1;15:444. doi: 10.1186/s12889-015-1792-4 (PMC4418047; doi:10.1186/s12889-015-1792-4)
Supplement: Additional file 1: — Original measurement items for child physical and emotional abuse victimisation. [file 12889_2015_1792_MOESM1_ESM.doc]

| Supplement 1: Questionnaire items for *Child physical and emotional abuse victimisation* | | |
| --- | --- | --- |
|  | **Questionnaire items:** | **Source:** |
| Physical Abuse | |  |
| How often in the past year did an adult… | |  |
|  | 1) use a stick, belt or other hard item to hit you? | UNICEF Measures |
|  | 2) slap, punch or hit you so that it hurt? | UNICEF Measures |
|  | 3) make you stand or kneel in an uncomfortable position for a long period of time to punish you? | Items design by local social workers, NGO staff, and children from the local community. |
| Emotional Abuse | |  |
| How often in the past year did an adult… | |  |
|  | 4) threaten to send you away or kick you out of the house? | UNICEF Measures |
|  | 5) threaten to invoke ghosts or evil spirits or harmful people? | UNICEF Measures |
|  | 6) call you dumb, lazy or other names? | UNICEF Measures |
|  | 7) withhold a meal to punish you? | UNICEF Measures |
|  | 8) single you out to do household chores all day instead of school or play? | UNICEF Measures |
|  | 9) threaten to hurt you or give you bad grades? | Items design by local social workers etc. |
|  | 10) insult members of your family that have passed away? | Items design by local social workers etc. |
|  | 11) tell you t hey wished they did not have to look after you or make you feel you are a burden | Item designed by local social workers etc. |
|  | 12) threaten to leave you and never come back? | Items design by local social workers etc. |
|  | 13) make you feel unwelcome at home? | Items design by local social workers etc. |
|  | 14) threaten to hurt or kill a person or an animal that you care about? | Items design by local social workers etc. |
