# Supplementary material for: Household illness, poverty and physical and emotional child abuse victimisation: findings from South Africa’s first prospective cohort study
Source: BMC Public Health. 2015 May 1;15:444. doi: 10.1186/s12889-015-1792-4 (PMC4418047; doi:10.1186/s12889-015-1792-4)
Supplement: Additional file 2: — Physical and emotional abuse at follow-up: Individual response frequencies. [file 12889_2015_1792_MOESM2_ESM.doc]

| Supplement 2: Physical and emotional abuse at follow-up: Individual response frequencies (n=3401) | | | | | |
| --- | --- | --- | --- | --- | --- |
| **Physical abuse:** | Never (n) | Not in the past year (n) | At least once this year (n) | Monthly (n) | Weekly (n) |
| use a stick, belt or other hard item to hit you? | 51.8% (1761) | 18% (613) | 16.9% (574) | 7.5% (254) | 5.8% (198) |
| slap, punch or hit you so that it hurt? | 76.1% (2589) | 7.6% (258 | 10.5% (356) | 4.1% (139) | 1.6% (56) |
| make you stand or kneel in an uncomfortable position for a long period of time to punish you? | 89.5% 3045 | 4.1% (138) | 4% (137) | 1.3% (44) | 1% (33) |
| **Emotional abuse:** | Never (n) | Not in the past year (n) | At least once this year (n) | Monthly (n) | Weekly (n) |
| threaten to send you away or kick you out of the house? | 91.4% (3107) | 1.1% (37) | 3% (103) | 2.6% (87) | 1.9% (64) |
| threaten to invoke ghosts or evil spirits or harmful people? | 96.9% (3296) | .5% (18) | 1.4% (47) | .8% (26) | .3% (11) |
| call you dumb, lazy or other names? | 80.1% (2724) | 2.1% (71) | 5.6% (190) | 5.1% (175) | 7% (238) |
| withhold a meal to punish you? | 94.6% (3216) | 1.% (35) | 1.9% (66) | 1.3% (45) | 1.1% (37) |
| single you out to do household chores all day instead of school or play? | 92.5% (3135) | 1.4% (49) | 2.6% (90) | 1.6% (55) | 2.1% (70) |
| threaten to hurt you or give you bad grades? | 95.3% (3240) | .9% (29) | 2.1% (73) | 1% (34) | .6% (21) |
| insult members of your family that have passed away? | 95.5% (3247) | .9% (32) | 1.7% (57) | 1.2% (40) | .6% (22) |
| tell you they wished they did not have to look after you or make you feel you are a burden | 94.6% (3217) | .9% (31) | 2.1% (72) | 1.3% (34) | 1% (35) |
| threaten to leave you and never come back? | 95.7% (3256) | .8% (27) | 1.3% (43) | 1.4% (46) | .8% (27) |
| make you feel unwelcome at home? | 86.2% (2930) | 1.4% (46) | 3.6% (123) | 3.7% (125) | 5.1% (173) |
| threaten to hurt or kill a person or an animal that you care about? | 96.9% (3296) | .7% (23) | 1.1% (36) | .7% (23) | .6% (21) |
